# Flashbulb memories in the context of group hierarchies: effects of gender, system justification, and social dominance orientation on negative private and public flashbulb memories

**DOI:** 10.3389/fpsyg.2024.1488241

**Published:** 2024-11-27

**Authors:** Merve Çavuşoğlu, Muharrem Ersin Kuşdil

**Affiliations:** Department of Psychology, Bursa Uludağ University, Bursa, Türkiye

**Keywords:** flashbulb memory, canonical category of flashbulb memory, phenomenological aspects of flashbulb memory, psychosocial functions flashbulb memory, gender differences in flashbulb memory, social dominance orientation, system justification tendency, group hierarchies

## Abstract

Research on flashbulb memories (FBMs) has primarily focused on cognitive aspects. However, recent studies indicate that FBMs are closely associated with social and cultural dynamics. This descriptive study explored the structural aspects and psychosocial functions of negative FBMs within the context of intergroup theories, mainly focusing on negative public (coup attempt in Türkiye on July 15, 2016) and private (bad news of a loved one) FBMs. Participants in the main study (*N* = 233) were selected and grouped based on their social dominance orientations (SDO; high and low groups) and system justification tendencies (SJT; high and low groups), using data from a preliminary survey (*N* = 1,113). In the main study, participants’ responses to items on canonical categories, phenomenological aspects, and psychosocial functions of FBMs and their involvement in different protest actions against the coup attempt were compared considering SDO, SJT, and gender. The results show that private FBMs were generally rated higher by participants than public FBMs in all aspects. Although the canonical quality of private FBM did not differ between gender groups, public FBM quality was better in men. Participants in the high-SJT group had higher-quality public FBMs and rated these memories as more functional than participants in the low-SJT group, along with their high levels of protest participation. No differences were observed between the SDO groups for either type of FBM. The findings are discussed in terms of their relevance to group hierarchies and system justification motives. As the first attempt to place the FBM phenomenon in the context of SDO and SJT at the individual level, this study is intended to encourage others to adopt multi-level intergroup theories for integrating bottom-up and top-down processes.

## 1 Introduction

Recently, flashbulb memories (FBMs), a type of autobiographical memory “of the circumstances in which one first learned of a very surprising and consequential (or emotionally arousing) event” (Brown and Kulik, 1977, p. 73), have been increasingly applied in studies examining processes related to collective memory. The mainstream social cognitive approach conceptualizes FBM formation and maintenance at the individual level (Demiray and Freund, 2015; Talarico and Rubin, 2018). However, a growing number of researchers consider FBMs to be on a memory continuum extending from the individual to the collective, especially those related to public events (Berntsen, 2018; Cheriet et al., 2023; Hirst and Meksin, 2018; Neisser, 1982). This renders FBM an indispensable component of both autobiographical and collective memory. According to this relatively recent perspective, people’s interactions with other members of society and mass media shape collective memories of FBM events. Events are represented from the perspective of the community, even though it is the individual who maintains them in his/her mind, and collective memories affect social identity through FBMs (Hirst and Meksin, 2018). Generally, as is widely acknowledged, an individual’s identity is partially constituted by their autobiographical memories (Conway, 2005), and collective identity is partly shaped by the shared memories of a given collective (Hirst and Manier, 2008).

FBMs fall in the middle of this taxonomy (Cheriet et al., 2023). Given their interpersonal diversity, FBMs cannot be viewed as purely collective memories. As Neisser (1982) noted, they represent situations in one’s life where personal and societal aspects intersect. FBMs have been studied in the context of collective memory because many members of affected communities report having FBMs of important public events, albeit with interpersonal variation in content (Hirst and Phelps, 2016). Such memories are the most prominent examples of the intersection between our past and societal history (Hirst and Phelps, 2016; Neisser, 1982). Moreover because the content of such memories corresponds to individuals’ different psychosocial needs (Demiray and Freund, 2015; Stone and Jay, 2018), FBMs will inevitably show considerable diversity throughout society. This diversity may be even higher in chronic or acute forms in some societies than in others, suggesting that FBMs can be examined through various social parameters, including social inequalities and legitimacy issues.

Given the recent developments in intergroup behavior theories, research on such social parameters at a theoretical level is now possible when exploring FBMs. Social identity theory, social dominance theory, and system justification theory are well-known examples of multilevel theories that can connect analyses on different levels ranging from the individual to societal (Çavuşoğlu and Kuşdil, 2023). Based on the assumption that when people form FBMs at a public event, they establish a connection between their experiences and the event itself (Hirst and Meksin, 2018), this study examined how the structural aspects and psychosocial functions of FBMs differ based on one’s system justification tendency (SJT), social dominance orientation (SDO), and gender in terms of event type. The tendency of participants to protest the coup was also examined in relation to SJT, SDO, and gender. This study was conducted with a sample of Turkish undergraduates, and their public (coup attempt) and private (hearing bad news about a close person) FBMs were used to examine how an individual’s perception of the social system and disposition affect FBMs of a public event. To the best of our knowledge, this descriptive study is the first attempt to locate the FBM phenomenon in the context of group hierarchies and systemic inequalities.

### 1.1 Characteristics of flashbulb memories

In addition to their event memory characteristics (Hirst et al., 2009, 2015), FBMs contain canonical properties that include vivid details (e.g., when one learned the event occurred, the person who provided information about the event, and one’s location and the activities carried out at that time) related to the context surrounding a person at the moment they learned the event had taken place (Brown and Kulik, 1977; Kaya-Kızılöz and Tekcan, 2013).

Although FBMs are as likely to be distorted or forgotten as ordinary autobiographical memories, some phenomenological characteristics make it possible to distinguish one from the other in terms of imagery and memory quality, such as surprise reactions, the importance given, emotional intensity, vividness, detail, confidence in the accuracy of the memory, and a certain level of rehearsal (Hirst and Phelps, 2016; Luminet and Curci, 2009; Talarico and Rubin, 2018). In the current literature, events that trigger FBMs are generally analyzed in two dimensions in terms of content: (1) types of events (private–public) and (2) emotional valence of the events (positive–negative). These two dimensions are asymmetrical, and negative public events have been analyzed more intensively than the other types in FBM research (Frinco et al., 2024). For example, collective and political violence, political crises, collective disasters, and presidential and celebrity deaths or assassinations often stand out in the research as events that exhibit FBM characteristics (for details of event examples, see Páez et al., 2018). However, positive public FBMs, such as political revolutions, sports victories, and political elections, have been examined in several studies (e.g., Bohn and Berntsen, 2007; Chiew et al., 2022; Kopietz and Echterhoff, 2014; Tinti et al., 2014).

Findings from this research stream have shown differences regarding emotional valence when comparing private and public FBMs. Pillemer (2009) found that private FBMs generally exhibit higher consequentiality, personal importance, and rehearsal than public FBMs. Between positive and negative public events, although Talarico and Rubin (2018) concluded that research on emotional valence produced mixed findings on consistency and confidence, results of a recent study by Raw et al. (2023) conducted in the UK on FBMs of the 2016 Brexit Referendum suggest that negativity enhances accuracy, whereas positivity creates overconfidence. Among Danes aged 72–89 years, Berntsen and Thomsen (2005) found that FBMs of events during World War II were clearer and more accurate when positive (liberation from the German invasion) than when negative (German invasion of Denmark); however, both FBMs were similar in vividness. Furthermore, whether an event is perceived as having a positive or negative valence may change based on the groups involved. For example, Bohn and Berntsen (2007) studied the political revolution of the 1989 fall of the Berlin Wall as an FBM event in a sample of German participants. Those who had lived in West Germany at the time interpreted the event positively and showed higher vividness but less accurate event memory compared with those who had lived in East Germany and interpreted the event negatively. In contrast, those who had lived in East Germany had higher scores for consequentiality but lower scores for rehearsal than those who had lived in West Germany.

### 1.2 Psychosocial functions of flashbulb memories

FBMs are common in individuals and large groups and are thus expected to serve essential psychosocial functions. Research has indicated that autobiographical memories serve at least three functions: self-continuity, social bonding, and directive (Bluck et al., 2005). The *self-continuity function* involves recalling autobiographical memories to stabilize or assess changes in the self, identity, values, and beliefs. The *social bonding function* preserves memories, facilitates new relationships, and maintains and deepens existing relationships. Some researchers propose that social bonding is the main function of autobiographical memory because these memories are used in conversations to establish closeness in relationships and transfer information (for a discussion, see Bluck and Alea, 2011). Finally, the *directive function* concerns how memories are recalled to guide actions to solve current problems and direct thoughts and behaviors to construct future goals.

In the first empirical study on the functions of FBMs, Rasmussen and Berntsen (2009) compared different types of memories, such as autobiographical memories, FBMs, and involuntary memories regarding their functions (self, social, and directive) and found that FBMs were considered more critical than other memories for social functioning. Furthermore, the social functions of FBMs were found to be more critical than the individual functions, such as self and behavior guidance. Moreover, participants were shown to share FBMs with others more frequently than other types of memories. Regarding emotional valence, negative memories were found to fulfill more directive functions than positive memories.

In a comprehensive study comparing the emotional valences of private and public FBMs, Demiray and Freund (2015) examined their psychosocial functions. After determining the content of memories in a preliminary study with an American sample, they compared positive (Osama Bin Laden’s death) and negative (Michael Jackson’s death) public FBMs with positive (learning of one’s own pregnancy or that of a loved one) and negative (learning of the illness, accident, or death of a loved one) private FBMs in terms of canonical categories, phenomenological aspects, and psychosocial FBM functions (i.e., self-continuity, social bonding, and directive functions). The findings revealed that private FBMs serve all three functions to a greater extent than public ones; however, private FBMs served a stronger social bonding function when positive rather than negative.

Some researchers have argued that these psychosocial functions differ according to the emotional valence of an FBM. For example, based on existing research on FBM functions, Stone and Jay (2018) suggested that positive FBMs, whether private or public, may be associated with social integration functions such as self-definition and psychological and social well-being. In contrast, negative FBMs may be associated with directive functions such as problem-solving and guiding future behavior. Similarly, Lind et al. (2019) suggested that negative autobiographical memories are more likely to fulfill directive functions than social or self-continuity functions.

### 1.3 Flashbulb memories in an intergroup context

FBMs are viewed as individual- and community-based phenomena, as they occur within a community. Elaborating on Neisser (1982, p. 48) well-known statement about individuals’ use of FBMs to express that “I was there,” Hirst and Meksin (2018, p. 202) stated that, through FBMs, an individual implicitly claims that “I was there with other members of the affected community.” Therefore, FBMs should be functional not only for individuals but also for society. Numerous studies have been based on the assumption that residents of the same country frequently have collective memories, which are shared, culturally molded recollections of important historical events (Burnell et al., 2022), and some examples show this is the case in several countries (Liu, 2022).

However, in some cases, individuals, groups, or societies may differ significantly in how they evaluate the same public event. For example, the election of Donald Trump as US President in 2016 was a public event that elicited extreme emotional reactions that were positive for some individuals in American society but negative for others (Chiew et al., 2022). In contrast, the killing of Osama Bin Laden was widely evaluated as a positive event in American society, although for some members of political and religious minorities, it stood out as a negative event (Demiray and Freund, 2015; Kraha et al., 2014). Similarly, FBMs related to the results of the 2016 referendum on the UK’s departure from the European Union were negative for those who voted to remain and positive for those who voted to leave (Raw et al., 2023). These findings suggest that intra-and cross-country diversity can provide essential contextual depth to consider when studying FBMs. Studies of “living historical memories” (LHMs), which “consist of historical events that are widely shared through everyday communication between people in society” (Liu et al., 2021, p. 105), also show that some societies may have no “master narrative” that binds individuals together. In their research on social media and dissident LHMs in Hong Kong, Li et al. (2021) found that historical events are associated with anti-government perspectives in places like Hong Kong, where a trend toward protesting Beijing rule is evident. In their study on legitimizing ideologies in post-Soviet democratic Estonia, Kus et al. (2013) found that ethnic Estonians, the current dominant group, and ethnic Russians, the former dominant group, reported using different historical aspects to support their perceptions of justice and dominance. However, in countries with a stable governmental system, such as the monarchy in Morocco, collective memories of protest events are associated more with support for the existing social system (Bou Zeineddine and Qumseya, 2021).

Several studies have highlighted the importance of social identity in FBM formation and maintenance (Berntsen, 2018; Cordonnier and Luminet, 2021; Hirst and Phelps, 2016; Merck and Hirst, 2022; Merck et al., 2020). For example, Berntsen (2018) suggested that, although activating a social identity may elicit an emotional response and rehearsal, which may explain individuals’ enhanced memory, the social relevance of public events should be prioritized over personal relevance. In studies where groups are compared, groups with some connection to the event tend to report more FBMs. For example, Morse et al. (1993) found that women reported more FBMs than men of learning about the appointment of Clarence Thomas, who was accused of sexual harassment, to the US Supreme Court, with women also having more vivid FBMs than men. However, no significant gender differences were found in the ratings given to these FBMs. However, Wright et al. (1998) showed that men had clearer and more vivid FBMs than women concerning the Hillsborough disaster, known as the worst accident in the history of soccer, despite men feeling that it was less important and emotional than women did.

### 1.4 SDO and system justification as basic mechanisms

The mechanisms underlying the role of social identity and group membership in maintaining FBMs are not yet fully understood. Building on previous studies, Griffiths et al. (2022) concluded that group members occupying higher positions in the social hierarchy recalled hierarchical information with fewer errors than low-status group members. However, few studies have attempted to explain why memories related to public events are more robust and functional in some groups than in others. A theoretical explanation may come from the concept of “ideological asymmetry” from Sidanius and Pratto’s (1999) social dominance theory. This concept suggests that psychological and ideological mechanisms that help maintain a group-based hierarchy operate more efficiently in dominant than in subordinate group members. For example, dominant group members can adopt hierarchy-enhancing myths (e.g., nationalism, sexism, and racism) more efficiently, and their motives to justify hierarchical structures exhibit high congruence with their SDOs, which is defined by Sidanius et al. (2016, p. 152) as “the general desire to establish and maintain hierarchically structured intergroup relations regardless of the position of one’s own group(s) within this hierarchy.” This high level of congruence makes it easier for advantaged groups to maintain their position in the social hierarchy because the congruence between attitudes and actions facilitates their ability to make decisions faster and take practical actions aligned with their interests. In contrast, members of subordinate groups experience difficulties demonstrating their power and determination to change their position in the status hierarchy owing to low congruence (Sidanius and Pratto, 1999). Therefore, social dominance theory refers to the high congruence observed in dominant group members between psychological processes, beliefs, attitudes, and behaviors that facilitate group-based dominance as ideological asymmetry. A limited number of studies support similar arguments regarding ideological asymmetry using samples from the US (Sidanius et al., 1996; Peña and Sidanius, 2002) and Canada (Fang et al., 1998; Lalonde et al., 2007).

Social dominance theory also proposes that the psychological outcomes of social hierarchies that produce economic surpluses can be analyzed by focusing on three basic hierarchical systems in which one group dominates the other: age (older people have power over younger), gender (men have power over women), and arbitrary-set systems (one group has power over others, depending on the historical/political conditions of any society). Combined with the ideological asymmetry explanation, this suggests that dominating groups use the advantage of having a more consistent attitude–behavior relationship in handling the social problems they encounter and thus maintain their privileged status in the social hierarchy (Sidanius and Pratto, 1999). Although social dominance theorists have not yet directly linked the concept of ideological asymmetry to memory processes, examining FBMs of public events through this asymmetry seems possible. If this asymmetry is valid, the FBMs of individuals with high levels of SDO for events that threaten the status quo would be shaped by the theme of domination and fulfill more psychosocial functions. However, it should be possible for low-SDO individuals’ FBMs of such events to be shaped and utilized in the opposite direction, linked to hierarchy-attenuating myths (e.g., social democracy and human rights).

Another explanation can be found in system justification theory, which posits, “system-justification refers to the psychological process whereby an individual perceives, understands, and explains an existing situation or arrangement with the result that the situation or arrangement is maintained” (Jost and Banaji, 1994, p. 10). Accordingly, the theory proposes that the motivation to legitimize the system amplifies individuals’ evaluations of the legitimization of social inequalities. This theory also suggests that social cognition serves a legitimizing function, whether explicit or implicit. Given that system-justification motivation is expected to increase, especially when the system is threatened (Kay and Friesen, 2011), individuals with high SJT would likely be more inclined to view public FBMs as supporting or threatening the system (Haines and Jost, 2000, pp. 222–223). In one of the few studies on this topic, Bonnot and Krauth-Gruber (2018) used experimental manipulation to make female participants feel dependent on the social system and found that they recalled autobiographical memories consistent with gender stereotypes (i.e., related to the verbal domain) more than memories related to the negative stereotype domain (i.e., scientific) and exhibited behavioral preferences (i.e., more verbal exercises than math exercises) in the same direction. In this study, the researchers also sought to distinguish between the effects of self-categorization and those of system justification. Accordingly, they exposed participants to a context that required legitimation without explicitly addressing gender issues or categorization. In light of these findings, the researchers posit that the need for women to perceive the system positively cannot be attributed to self or in-group favoritism alone.

While multilevel theories would clearly be useful in analyzing the FBM phenomenon, such studies are rare in related literature. Liu et al. (2021) covered 40 countries (not including Türkiye due to missing data) and focused on LHMs, finding that the number of such memories strongly correlated with lower SDO and lower system justification. Together with the finding that the demand for group equality is a universal theme in LHM, this supports the construction of national identity, especially in developing countries aiming for national progress. However, LHMs are also related to criticisms of these systems. Starting from the assumption that less-developed countries can be seen as “low-status groups in the global system,” Liu et al. (2021, p. 106) predicted that SDO and hierarchy-enhancing ideologies such as conservatism would correlate highly in developed societies rather than less developed ones. Building on the ideological asymmetry hypothesis, this logic suggests that the number of LHMs and legitimizing ideologies will show negative relationships in less developed countries. However, FBMs, which constitute a significant proportion of LHMs, have yet to be examined at the individual level or concerning their functions in relation to SDO and SJT.

### 1.5 The present study

In the present study, based on the view that SDO and SJT may be important for FBM emergence and maintenance, we examined how the structural characteristics and functions of individuals’ private and public FBMs with high and low levels of these variables differ in a sample from Türkiye, a developing country. Two studies were conducted for this purpose: a preliminary study with a large sample from undergraduates to identify participants with highest and lowest SDO and SJT tendencies, followed by the main study that compared selected participants in terms of the structural characteristics (canonical and phenomenological) and functions of FBMs depending on the event type (public or private) through groups formed according to varying SDO and SJT levels (high and low) and gender.

The coup attempt in Türkiye on July 15, 2016, was chosen as an example of a public FBM, and the news of a close person’s illness, accident, or death, which is known to be the most common negative life experience, was chosen as the private FBM. FBMs of the July 15 coup attempt have been previously addressed in only one social cognition study (Çavuşoğlu et al., 2021), which showed that this event can be examined as an FBM in terms of its canonical and phenomenological aspects. The private FBM was selected as a life event with negative emotional valence. Other studies have shown that receiving bad news about significant others has the characteristics expected of a negative private FBM (e.g., Demiray and Freund, 2015; Lanciano et al., 2018a; Niedźwieńska, 2003).

The July 15 coup attempt differs in some ways from previous coups in Türkiye (1960 and 1980). First, this attempt, which was quickly suppressed, started at a different time of day from the previous coups. While the previous coups started early in the morning, when everyone was asleep, this attempt started in the evening, when most people were going about their everyday lives. Another critical difference is that while previous attempts did not involve mass protests, a few hours after the start of this attempt, widespread protests and resistance took place in many cities after the president at the time called for them on television. This attempt, which was suppressed before dawn and left more than 250 people dead, generated different and contradictory reactions both socially and politically. A significant portion of the public saw the coup attempt as an attack on the AKP government, which had been in power for nearly 15 years. Other reactions generally believed that the coup attempt produced results that favored the ruling administration. Soon after the coup attempt, the government declared July 15^th^ an official holiday for the remembrance of the coup attempt as a threat to democracy. Therefore, this is an appropriate example of a negative public FBM in terms of the expected structural characteristics and its potential to generate varying individual reactions.

This study examined participants’ FBMs within the categories introduced above (gender, high and low SDO, and SJT) through canonical properties, phenomenological aspects (importance, consequentiality, emotional intensity, surprise, vividness, rehearsal, and emotional valence), and psychosocial functions (self-continuity, social bonding, and directive functions) prominent in the FBM literature. This study also examined the level of participation in protests against coup attempts and to determine the extent to which this is consistent with individuals’ SDO and SJT. The participants were also compared by gender, as some studies have reported gender differences in the phenomenological aspects of private and public FBMs. For example, in one study, American undergraduates were asked to remember the start of Operation Desert Storm (public event) and receiving the news of their acceptance into university (private event) (Tekcan, 2001). The participants generally reported stronger emotional reactions to private rather than public FBMs. Gender differences were also found in emotional reactions and the frequency of rehearsal; women showed stronger emotional reactions, albeit only to the news of Desert Storm, and more frequent rehearsals than men for both events. However, no gender differences were reported in participants’ recollection of details. In another study examining FBM content (vivid memories) in Polish participants aged 45–60 years, Niedźwieńska (2003) found that women’s narratives about vivid memories contained more communal themes (e.g., related to help, care, and understanding between people) and episodic autobiographical memory details (e.g., more emotional information, emotional intensity, consequentiality, and personal importance), whereas men’s narratives contained more competition, power, and prestige themes. However, the number of public events remembered did not differ by gender.

We posit that intergroup theories may offer a theoretical framework to explain such gender differences. For example, social dominance theory proposes a separate gender-set system in which men dominate women through several means, including violence (Sidanius and Pratto, 1999). Furthermore, social dominance theory asserts that young men are the main targets of violence and domination in an arbitrary system. As mentioned above, system justification motives are closely related to gender-based systemic inequalities. Given that the coup attempt was carried out against a government known to take a more traditional position on gender issues, it can be argued that the gender variable is worth examining in such a descriptive study. The present study used samples of undergraduates to address the following research questions (RQ):

RQ1: Do individuals’ private and public FBMs differ in terms of their canonical category properties, phenomenological aspects, and psychosocial functions in Turkish context?

RQ2: Do the phenomenological aspects and psychosocial functions of private and public FBMs and the frequency of their participation in protest actions against the coup attempt differ among individuals with high and low SDO and SJT as well as between genders?

## 2 Materials and methods

### 2.1 Preliminary study

#### 2.1.1 Participants and procedure

The preliminary study collected data from 1,282 juniors and sophomores by administering a questionnaire in university classrooms. Data from 130 participants (10.1%) who did not complete the forms correctly, nine of whom were foreign nationals (0.7%), and 30 participants (2.4%) who were over 25 years old were excluded from the sample. The final sample included in the data analysis included 1,113 undergraduates aged 18–25 years (*M*_age_ = 20.05, *SD*_age_ = 1.54; 668 female, 444 male, and one unspecified gender). Most participants resided in metropolitan areas (61%) and had a middle socioeconomic status (63%). Participants’ political orientations were distributed as follows: left (15.3%), center (38.8%), and right (10.1%).

The questionnaires consisted of the SDO and SJT scales and questions on sociodemographic information. The SDO and SJT scales were administered in alternating order. The participants were informed that the study consisted of two phases, and they could provide contact information on a separate page of the data collection form if they wanted to participate in the second study. The participants were also informed that five people who completed both studies would each be given a gift voucher of 100 Turkish Liras. The questionnaires took approximately 30–35 min to complete and were administered in the classrooms of various faculties of Bursa Uludağ University in Türkiye. Among the students, 12.7% did not volunteer to participate in the main study.

#### 2.1.2 Instruments

##### 2.1.2.1 System justification tendency

SJT was measured with the Turkish version (Yıldırım and Akgün, 2013) of the System Justification Scale developed by Kay and Jost (2003) for assessing the situational effects on the extent to which a person perceives the current social structure as fair (e.g., “Society is set up so that people usually get what they deserve”), legitimate (e.g., “In general, the Turkish political system operates as it should”), and justifiable (e.g., “Türkiye is the best country in the world to live in”). Participants rated their opinions on each statement using a five-point Likert scale ranging from 1 (*strongly disagree*) to 5 (*strongly agree*). The one-factor structure of this eight-item instrument was tested in the present study using confirmatory factor analysis (CFA). Fit indices indicated that the data fit the one-factor model well after adding two covariates between error terms (*χ^2/20^* = 1.94, *p* = 0.01, RMSEA = 0.029, CFI = 0.99). In this study, Cronbach’s alpha was 0.83.

##### 2.1.2.2 Social dominance orientation

Participants’ SDO levels were measured using the Turkish adaptation of Ho et al.’s (2015) SDO_7(s)_ (Kablanoğlu and Kuşdil, 2020). The scale comprises two dimensions: dominance and anti-egalitarianism. Anti-egalitarianism is the desire to support hierarchical relations and build a social system on inequality, even if it is to the detriment of one’s ingroup (e.g., “It is unjust to try to make groups equal”). Group-based dominance reflects the desire for one’s ingroup to climb the social hierarchical structure (e.g., “Some groups of people are simply inferior to other groups”). Participants rated their opinions on each statement on a seven-point Likert scale ranging from 1 (*strongly oppose*) to 7 (*strongly favor*). The fit indices of the CFA indicated that the two-factor model generally fit the data well (*χ^2/14^* = 3.68, *p* < 0.001, RMSEA = 0.05, CFI = 0.97, SRMR = 0.03). In this study, Cronbach’s alpha was 0.72 for the whole scale.

##### 2.1.2.3 Demographics

The last part of the questionnaire included 11 demographic questions about the respondents’ age, gender, department, socioeconomic level, the importance they attached to their social identity, political orientation, and level of following and talking about the country’s political agenda. Participants were asked to indicate their political orientation on a seven-point scale ranging from ±3 (*extreme, moderate, mild*) from left to right. Socioeconomic level (1 = *lower*; 5 = *higher*), importance attached to the participant’s social identity (1 = *not at all*; 5 = *very efficient in defining my social identity*), and interest in following and talking about the country’s political agenda (1 = *never*; 5 = *very frequently*) were measured using a 5-point Likert scale.

### 2.2 Main study

#### 2.2.1 Participants and procedure

Participants were identified using the median scores of SDO and SJT and divided into two groups according to their scores: low/high SDO (*Mdn =* 2.63) and low/high SJT (*Mdn =* 2.13). Invitations were sent to 327 participants among those who volunteered to participate in the study and met the criteria. Data from five participants reported not having private FBM and one participant who had missing data was excluded from the sample. Finally, a total of 233 participants aged 18–24 years (*M_age_* = 20.04, *SD_age_* = 1.57), including 146 women (62.7%) and 87 men (37.3%) participated in the main study (see, Table 1).

**Table 1:** Characteristics of the main study sample.

	Gender					
	Female *n*	Male *n*	Total *N*	*M_age_* (*SD_age_*)	*M_SDO_* (*SD_SDO_*)	*M_SJT_* (*SD_SJT_*)
Low SDO	88	31	119	19.86 (1.53)	1.60 (0.44)	1.94 (0.66)
High SDO	58	56	114	20.24 (1.59)	3.83 (0.77)	2.16 (0.82)
Total	146	87	233			
Low SJT	84	54	138	20.05 (1.54)	2.58 (1.29)	1.52 (0.28)
High SJT	62	33	95	20.03 (1.61)	2.86 (1.25)	2.81 (0.52)
Total	146	87	233			

The first part of the questionnaire included canonical category questions, phenomenological aspects, psychosocial functions of public FBMs, and frequencies of actions related to protesting the coup attempt. The second part included canonical category questions, phenomenological aspects, and psychosocial functions of private FBM. Four attention-check questions were added among the private and public FBM phenomenological aspects and psychosocial functions scales (e.g., “For technical reasons, please answer this question by checking ‘often’”) to determine whether the participants followed the instructions appropriately. Public FBM questions were always answered first, and private FBM questions were answered second. The second part included an additional question for private events: “What news you received?” This question was used only to check the content of the events. Data were collected through individual applications in the psychology department, which took an average of 20–25 min. The data were collected approximately 35 months after the coup attempt.

#### 2.2.2 Instruments

##### 2.2.2.1 Canonical category questions

The canonical categories for addressing the elements of the reception context of participants’ FBMs were assessed using five questions adapted from various studies (e.g., Demiray and Freund, 2015; Gandolphe and El Haj, 2017; Kvavilashvili et al., 2010). The first was a closed-ended question asking the participants whether they remembered the FBM event (private and public), to which they could answer “yes” or “no.” None of the participants answered “no” to this question for the public FBM; however, five participants stated they did not have a private FBM of the type specified in the instructions. The data from these participants were not included in the analyses. The other four questions were open-ended and related to how and from whom the event was learned, their location and time when they learned of the event, and the activity in which they were engaged at the time. Participants answered these questions by writing in the text boxes allocated for each question. If they had difficulty remembering, they were told to answer these questions by checking either “I do not remember” or “I do not know” in each text box.

##### 2.2.2.2 Phenomenological aspects of FBMs

This study included a 14-item scale consisting of questions about the aspects of recalled FBMs. This scale was developed by Çavuşoğlu (2021) based on a review of the relevant literature (e.g., Berntsen and Thomsen, 2005; Gandolphe and El Haj, 2017; Kvavilashvili et al., 2010). Questions were included on the following: (1) importance of the event for the individual and (2) for society, (3) emotional intensity, (4) visual relieving of the event while remembering it, (5) physiological reaction, (6) mental travel in time, (7) clarity, (8) thinking about the event, (9) talking about the event, and (10) frequency of following the news through social and mainstream media, (11) level of surprise, (12) emotional valence of the event, and (13) short-and (14) long-term consequentiality (for details of questions, see Table 2). The importance variable was presented for the participants to rate on a 10-point Likert scale ranging from 1 (*not at all important*) to 10 (*very important*). The emotional valence variable was rated on a 5-point Likert scale ranging from −2 to 2 (*very negative*–*very positive*). The other 14 statements were randomly ordered and presented to the participants with five-point Likert scales ranging from 1 (*strongly disagree*) to 5 (*strongly agree*).

**Table 2:** Questions asked to measure the phenomenological aspects of FBMs.

Variable	Item	Authors	Factor that item was loaded in this study
Personal importance	When you first heard [event in question], how important was if for you personally?	Kvavilashvili et al. (2010)	Importance
National importance	How important was [event in question] considered in Türkiye?	Kvavilashvili et al. (2010)	
Visual relieving	When you remember the moment when you first learned about [event in question], do you see this moment in your mind?	Gandolphe and El Haj (2017)	Vividness
Clarity	When you remember the moment when you first learned about [event in question], do you remember it quite as clearly as if it happened now?	Berntsen and Thomsen (2005)	
Physiological reaction	When you remember the moment when you first learned about [event in question], do you feel any physiological reaction (e.g., accelerated heartbeat, nervousness, sweating, feeling like crying)?	Gandolphe and El Haj (2017)	
Mental travel in time	When you remember the moment when you first learned about [event in question], do you feel that you travel back to the time it happened?	Gandolphe and El Haj (2017)	
Frequency of thinking about the event	Since its announcement, how often have you by yourself thought about [event in question]?	Gandolphe and El Haj (2017)	Rehearsal
Frequency of talking about the event	Since its announcement, how often have you talked to others about [event in question]?	Gandolphe and El Haj (2017)	
Frequency of following the news in mainstream and social media	Since the announcement of [event in question], how closely have you followed the mainstream media and social media coverage and discussions?	Gandolphe and El Haj (2017)	
Short-term consequentiality	How many immediate changes did [event in question] cause for you and your surroundings when it took place?	Berntsen and Thomsen (2005)	Consequentiality
Long-term consequentiality	How many long-term consequences did [event in question] have for you and your life?	Berntsen and Thomsen (2005)	
Surprise	When you first learned about [event in question] how surprising was it?	Gandolphe and El Haj (2017)	Surprise
Emotional valence	How emotionally positive/neutral/negative did [event in question] appear to be when it took place?	Berntsen and Thomsen (2005)	Emotional valence
Emotional intensity	How intense were your emotions when [event in question] took place?	Berntsen and Thomsen (2005)	Emotional intensity

This scale, which was previously tested using CFA in a general sample in Türkiye (Çavuşoğlu et al., 2021), has a seven-factor structure. The same factor structure was tested again using two CFAs for public and private FBMs in the present study. The CFA results for the public FBM showed that the seven-factor model fit the data well (*χ^2/55^* = 1.94, *p* = 0.001, RMSEA = 0.06, CFI = 0.96, SRMR = 0.06). Similarly, the fit indices derived from the CFA results for private FBMs indicated that this model also fit the data well (*χ^2/55^* = 1.41, *p* = 0.00, RMSEA = 0.04, CFI = 0.99, SRMR = 0.03). The results of the reliability analyses for the FBM subscales, most of which contained few items, also revealed acceptable standardized Cronbach’s alpha coefficients for both types of FBMs: 0.61 for vividness (3 items); 0.79 for importance (2 items); 0.74 for rehearsal (2 items); and 0.77 for consequentiality (2 items) for Public FBM. Private FBM Cronbach’s alpha coefficients were 0.76 for vividness (4 items), 0.59 for rehearsal (2 items), and 0.83 for consequentiality (2 items). Surprise, emotional valence, and emotional intensity were measured using a single item.

##### 2.2.2.3 Psychosocial functions of FBM

The psychosocial functions of private and public FBMs were measured using the Turkish adaptation (Göz, 2016) of the revised Thinking about Life Experiences Questionnaire–TALE (Bluck and Alea, 2011). The scale has 15 total statements, with the following subscales each having five statements: of self-continuity (e.g., “When I want to feel that I am the same person that I was before”), social bonding (e.g., “When I also hope to find out what another person is like”), and directive (e.g., “When I believe that thinking about the past can help guide my future”). The subscales were presented on a five-point Likert scale ranging from 1 (*almost never*) to 5 (*very frequently*).

The three-dimensional structure of the original TALE was tested separately for public and private FBMs using two separate CFAs. After removing two items (“When I want to feel that I am the same person that I was before” and “When I am concerned about whether my beliefs have changed over time”) from the self-continuity subscale, two items (“When I also hope to find out what another person is like” and “When I want to maintain a friendship by sharing memories with friends”) from the social bonding subscale, and one item (“When I need to make a life choice and am uncertain which path to take”) from the directive subscale for the public FBM, the fit indices showed that the three-factor structure fit the data well (*χ^2/87^* = 2.20, *p* = 0.0001, RMSEA = 0.07, CFI = 0.96, SRMR = 0.04, GFI = 0.93).

After removing two items (“When I also hope to find out what another person is like” and “When I also hope to learn more about another person’s life”) from the social bonding subscale and one item (“When I want to try to learn from my past mistakes”) from the directive subscale for private FBMs, the fit indices indicated that the three-factor structure fit the data well (*χ^2/87^* = 1.83, *p* = 0.001, RMSEA = 0.06, CFI = 0.98, SRMR = 0.03, GFI = 0.96). Cronbach’s alpha coefficients for self-continuity, social bonding, and directive subscales were 0.82, 0.80, and 0.85 for public FBMs and 0.87, 0.83, and 0.77 for private FBMs, respectively.

##### 2.2.2.4 Frequency of participation in the protest actions of the coup attempt

An instrument was developed to measure the frequency of participation in activities organized to protest the coup attempt (e.g., participating in protest marches organized after the attempt and posting on social media). Seven questions were presented to participants on a five-point Likert scale ranging from 1 (*never*) to 5 (*very frequently*). The last question was open-ended and asked participants whether they had taken any other actions in protest. The exploratory factor analysis results of the data derived from the responses to the seven items indicated the presence of a single factor with an eigenvalue greater than 1 (3.31), explaining approximately half of the variance (47.3%). In addition, all items loaded on this factor had loadings greater than 0.40. The one-factor model was then tested using CFA. The results revealed that the model fit the data well after adding covariance between the two error terms (*χ^2/14^* = 1.61, *p* = 0.08; RMSEA = 0.05; CFI = 0.99; SRMR = 0.04; GFI = 0.98). In this study, Cronbach’s alpha was 0.81 for all items.

## 3 Results

### 3.1 Preliminary analyses

#### 3.1.1 Sociodemographic characteristics of the main study sample

In the first step, we conducted separate one-way analyses of variance (ANOVA) to check for significant differences between the SDO and SJT scores of the high/low SDO, high/low SJT, and gender categories (see Table 1 for means and standard deviations). The results showed that means of the high SDO group (*M* = 3.83, *SD* = 0.77) were significantly higher than those of the low SDO group (*M* = 1.60, *SD* = 0.44) for SDO scores, *F*_1, 231_ = 742.14, *p* < 0.001, *𝜂_p_^2^* = 0.76. Similarly, the high SDO group (*M* = 2.16, *SD* = 0.82) had significantly higher means than the low SDO group (*M* = 1.93, *SD* = 0.66) for SJT scores, *F*_1, 231_ = 5.28, *p* < 0.05, *𝜂_p_^2^* = 0.02. Furthermore, the high SJT group (*M* = 2.81, *SD* = 0.52) had higher mean scores than the low SJT group (*M* = 1.52, *SD* = 0.28) for SJT, *F*_1, 231_ = 601.02, *p* < 0.001, *𝜂_p_^2^* = 0.72. No significant differences were observed between SJT groups for SDO scores, *F*_1, 231_ = 2.64, *p* = 0.11. The results of the ANOVA for SDO and SJT scores by gender showed that men (*M* = 3.23, *SD* = 1.32) had higher SDO scores than women (*M* = 2.37, *SD* = 1.14), with a significant difference *F*_1, 231_ = 27.68, *p* < 0.001, *𝜂_p_^2^* = 0.11. However, no significant gender differences were observed for SJT scores, *F*_1, 231_ = 0.59, *p* = 0.44.

Secondly, one-way ANOVAs were conducted to analyze differences in demographic variables, including socioeconomic status, political orientation, attachment to social identity, following the political agenda, and discussing the political agenda in the SDO, SJT, and gender groups. Socioeconomic status was not significant in the high/low SDO (*F*_1, 230_ = 0.93, *p* = 0.34), high/low SJT (*F*_1, 230_ = 2.58, *p* = 0.11) or gender groups (*F*_1, 230_ = 3.79, *p* = 0.053). However, political orientations^1^ differed across SDO groups*, F*_1, 216_ = 14.35, *p* < 0.001, *𝜂_p_^2^* = 0.06. The high SDO group (*M* = 4.02, *SD* = 1.35) were found to have more right-wing political views than the low SDO group (*M* = 3.35, *SD* = 1.22). Political orientations also differed between the SJT groups, *F*_1, 216_ = 57.33, *p* < 0.001, *𝜂_p_^2^* = 0.21. Specifically, the high SJT group (*M* = 4.41, *SD* = 1.12) has more right-wing political views than the low SJT group (*M* = 3.18, *SD* = 1.22). But political orientation was not significant in gender groups (*F*_1, 216_ = 2.15, *p* = 0.14).

In addition, one-way ANOVAs were used to analyze the significance of social identity among the participants regarding group variables but none of them was significant (SDO: *F*_1, 227_ = 0.12, *p* = 0.74; SJT: *F*_1, 227_ = 3.69, *p* = 0.056, and gender: *F*_1, 227_ = 0.10, *p* = 0.92). Furthermore, the results of the separate one-way ANOVAs for the variables of “following and discussing the political agenda” indicated significant differences between the high and low SDO groups: the low SDO group (*M_following_* = 2.44, *SD_following_* = 1.07; *M_discussing_* = 1.87, *SD_discussing_* = 1.06) scored higher than the high SDO group (*M_following_* = 2.11, *SD_following_* = 1.16; *M_discussing_* = 1.55, *SD_discussing_* = 0.99) in both the following (*F*_1, 230_ = 5.10, *p* < 0.05, *𝜂_p_^2^* = 0.02) and discussing the agenda with others (*F*_1, 229_ = 5.63, *p* < 0.05, *𝜂_p_^2^* = 0.02). No significant differences were found based on SJT levels or gender.

#### 3.1.2 Determining the quality of FBMs

Canonical category questions were analyzed separately for private and public events. The quality of private and public FBMs was determined using scoring criteria prepared for the present study and analyzing four main canonical categories: how the participants learned about the event, location at the time of learning the event, the time of learning about the event, and the activity the participants were engaged in at the time of learning about the event. Two independent coders analyzed the participants’ responses to these questions. A score of “0″ was assigned to participants for their imprecise answers, such as “I do not remember” or inconsistency in the information provided for the event conditions, and a score of “1″ if the information provided was detailed, convincing, and directly related to the question. Subsequently, an index was created on a four-point scale by summing the coding of the responses to the four questions to determine how strong the participants’ FBMs were individually. Accordingly, when a participant gave ambiguous answers to all four canonical category questions, the participant was categorized as having “no FBM.” Participants who received a recall score of 1, 2, or 3 were categorized as having a “weak FBM.” Finally, participants who received a score of 4 were categorized as having a “strong FBM” and assumed to remember canonical categories better than those with weak FBMs (Demiray and Freund, 2015).

Five participants reported not having private FBMs of the type specified in the instructions. An analysis of the content of the participants’ private FBMs showed that 40.2% had received news of the death of a family member or close friend, 10.5% had received news of illness, and 8% of them received news of an accident (e.g., traffic accident, work accident, fire). Five participants (2.1%) did not share the content of their FBMs because it was too private for them to disclose. When the canonical categories for private FBMs were analyzed, only one participant responded with “I do not remember” (0.4%) for the source from which they learned about the event, five people (2.1%) for the identification of the location, 65 people (27.9%) for the identification of time, and 41 people (17.1%) for the identification of the activity. At the end of the coding process, Cohen’s kappa coefficients were calculated for each canonical category for private FBM, and the sum of the four categories indicated a good level of agreement between coders: 0.80 for source, 0.91 location, 0.94 for time, 0.93 for ongoing activity, and 0.94 for private FBM quality. Based on the classification, 130 participants (55.8%) were placed in the “weak private FBM” group and 103 (44.2%) in the “strong private FBM” group.

Regarding public FBMs, all participants reported remembering the events of the night of July 15, 2016. When the canonical categories were analyzed, responses of “I do not remember” were zero for the location where they learned about the event, one (0.4%) for the determination of the source, 60 (25.1%) for the determination of time, and 27 (11.3%) for the determination of the activity. Cohen’s kappa coefficients were calculated for each canonical category for public FBM, and the sum of the four categories indicated a good level of agreement between the coders: 0.93 for source, 0.98 for location, 0.82 for time, 0.89 for ongoing activity, and 0.95 for public FBM quality. Based on this categorization, 153 (65.7%) participants were placed in the “weak public FBM” group and 80 (34.3%) in the “strong public FBM” group. An exact McNemar’s test assessing FBM quality regarding canonical category groups (weak and strong) across different event types (private and public) showed a statistically significant difference in the quality of these two event types in terms of canonical categories, *p* = 0.02; the quality of individuals’ private FBMs was higher than that of their public FBMs^2^.

Then, chi-square tests were used to assess the associations between the quality of FBM regarding event type and high and low SDO, high and low SJT, and gender. The results showed no significant association between SDO and public [*χ^2^* (1, *N* = 233) = 0.62, *p* = 0.43] or private FBM quality [*χ^2^* (1, *N* = 233) = 0.18, *p* = 0.67]. Similarly, no significant associations were found between SJT and public FBM quality (*χ^2^* (1, *N* = 233) = 0.45, *p* = 0.50). The quality of the public FBM was high in both SJT groups [*χ^2^* (1, *N* = 233) = 4.62, *p* < 0.05]. Finally, a statistically significant, albeit small-sized, association was found between public FBM quality and gender, *χ^2^* (1, *N* = 233) = 6.78, *p* < 0.01, *φ* = 0.01: Men’s public FBMs had higher quality than those of women. No significant association was found between private FBM quality and gender, *χ^2^* (1, *N* = 233) = 2.28, *p* = 0.13 (for all frequencies, see Table 3).

**Table 3:** Chi-square frequency results.

		Gender		SJT		SDO		FBM Quality
		Female *f*	Male *f*	Low *f*	High *f*	Low *f*	High *f*	Total *f*
Public FBM	Weak	105	48	93	60	81	72	153
	Strong	41	39	45	35	38	42	80
	Total	146	87	138	95	119	114	233
Private FBM	Weak	87	43	85	45	68	62	130
	Strong	59	44	53	50	51	52	103
	Total	146	87	138	95	119	114	233

*N = 233.*

### 3.2 Phenomenological aspects of the FBMs

First, phenomenological aspects of the private and public FBMs were analyzed through one-way Repeated ANOVAs. Results showed that private FBMs exhibited higher quality in all dimensions except “surprise.” Participants’ frequency of rehearsal for private FBMs was higher than that for public FBMs (*F*_1, 232_ = 145.87, *p* < 0.001, *𝜂_p_^2^* = 0.39), which they remembered them more vividly (*F*_1, 232_ = 92.28, *p* < 0.001, *𝜂_p_^2^* = 0.29), assigned them more importance (*F*_1, 232_ = 66.80, *p* < 0.001, *𝜂_p_^2^* = 0.22), saw them as more consequential for their lives (*F*_1, 232_ = 161.10, *p* < 0.001, *𝜂_p_^2^* = 0.41), were more emotionally intense (*F*_1, 232_ = 174.71, *p* < 0.001, *𝜂_p_^2^* = 0.43), and that private FBMs had more negative than public FBM (*F*_1, 232_ = 134.98, *p* < 0.001, *𝜂_p_^2^* = 0.37). However, public FBMs were rated as more surprising than private FBMs (*F*_1, 232_ = 6.85, *p* < 0.01, *𝜂_p_^2^* = 0.03). The results of these analyses are provided in Table 4.

**Table 4:** Means and standard deviations of the phenomenological aspects and psychosocial functions of public and private FBMs.

	Private FBM		Public FBM		
Variables (Range)	*M*	*SD*	*M*	*SD*	*F*
Phenomenological aspects					
Rehearsal (1–5)	3.07	0.95	2.23	0.77	145.87**
Vividness (1–5)	3.98	0.80	3.38	0.75	92.28**
Importance (1–10)	9.30	1.33	8.06	1.98	66.80**
Consequentiality (1–5)	3.64	1.02	2.45	1.00	161.10**
Surprise (1–5)	3.97	1.13	4.18	0.78	6.85*
Emotional valence (±2)	−1.77	0.58	−1.09	0.84	134.98**
Emotional intensity (1–5)	4.51	0.73	3.53	0.94	174.71**
Psychosocial functions					
Self-continuity (1–5)	2.75	1.02	1.88	0.93	138.69**
Social bonding (1–5)	2.26	1.11	1.63	0.82	67.61**
Directive (1–5)	2.62	0.96	1.86	0.91	103.12**

*N* = 233. *p < 0.01, **p < 0.001.

Scores of the seven phenomenological dimensions for each event type in the SDO, SJT, and gender groups were analyzed using three separate 2×2 Mixed-Design ANOVAs. SDO (high/low), SJT (high/low), or gender (female/male) categories were added separately as between-subject factors in each analysis. Event type (public/private) was used as a within-subject factor for phenomenological aspects of each event (rehearsal, vividness, importance, consequentiality, surprise, emotional valence, and emotional intensity). Phenomenological dimensions for each event type in the SDO groups were analyzed using 2 (public/private) x 2 (high/low SDO) Mixed-Design ANOVAs. None of the phenomenological aspects of private and public FBMs differed significantly between the high-and low-SDO groups (0.13 < *p*’s < 0.91).

On the other hand, 2 (public/private) × 2 (high/low SJT) Mixed-Design ANOVAs revealed significant results in the phenomenological aspect dimensions between the high and low SJT groups, with small effect sizes. While no differences were found between the high and low SJT groups concerning importance given to private FBMs, the importance given to public FBMs was significantly higher in the high SJT group compared to the low SJT group (*F*_1, 231_ = 4.89, *p* < 0.05, *𝜂_p_^2^* = 0.02). Similarly, participants in the high SJT group reported that the public FBM was more consequential for their lives compared to that reported by the low SJT group (*F*_1, 231_ = 6.15, *p* < 0.05, *𝜂_p_^2^* = 0.03). However, this effect was not observed for private FBM. Although the public FBMs were remembered more negatively in the high SJT group than in the low SJT group, the emotional valence of the private FBMs did not differ between these groups (*F*_1, 231_ = 4.94, *p* < 0.05, *𝜂_p_^2^* = 0.02). The results for rehearsal (*F*_1, 231_ = 1.56, *p* = 0.21), vividness (*F*_1, 231_ = 0.26, *p* = 0.61), surprise (*F*_1, 231_ = 1.63, *p* = 0.20), and emotional intensity (*F*_1, 231_ = 0.76, *p* = 0.38) were not significant. The results of these analyses are provided in Table 5.

**Table 5:** Means and standard deviations of the phenomenological aspects and psychosocial functions of FBMs in SDO, SJT, and gender groups.

		SDO					SJT					Gender				
		Low SDO *n* = 119		High SDO *n* = 114			Low SJT *n* = 138		High SJT *n =* 95			Female *n* = 146		Male *n* = 87		
Variables (Range)	Event type	*M*	*SD*	*M*	*SD*	*F*	*M*	*SD*	*M*	*SD*	*F*	*M*	*SD*	*M*	*SD*	*F*
Phenomenological aspects																
Rehearsal (1–5)	Public	2.22	0.74	2.24	0.81	1.56	2.06	0.74	2.48	0.75	1.56	2.24	0.75	2.22	0.80	12.23***
	Private	3.15	0.95	2.99	0.95		2.97	0.95	3.22	0.94		3.26	0.91	2.75	0.94	
Vividness (1–5)	Public	3.41	0.75	3.34	0.74	2.34	3.33	0.77	3.45	0.71	0.26	3.38	0.74	3.37	0.76	3.14
	Private	4.11	0.74	3.85	0.84		3.90	0.82	4.09	0.75		4.07	0.78	3.83	0.82	
Importance (1–10)	Public	8.12	1.82	8.00	2.14	0.08	7.78	2.04	8.48	1.83	4.89*	8.19	1.87	7.85	2.15	0.25
	Private	9.40	1.26	9.19	1.39		9.29	1.45	9.32	1.14		9.37	1.26	9.18	1.43	
Consequentiality (1–5)	Public	2.39	0.94	2.53	1.06	1.45	2.32	0.96	2.65	1.03	6.15*	2.43	0.93	2.49	1.11	4.76*
	Private	3.68	1.04	3.59	1.01		3.69	1.04	3.55	1.00		3.77	1.01	3.41	1.02	
Surprise (1–5)	Public	4.23	0.79	4.14	0.77	0.04	4.16	0.76	4.22	0.81	1.63	4.21	0.69	4.14	0.92	0.41
	Private	3.99	1.15	3.94	1.12		4.03	1.11	3.87	1.16		3.95	1.05	3.99	1.26	
Emotional valence (±2)	Public	−1.13	0.80	−1.04	0.87	0.01	−0.99	0.81	−1.23	0.86	4.94*	−1.13	0.81	−1.01	0.88	0.30
	Private	−1.82	0.56	−1.71	0.59		−1.78	0.60	−1.76	0.54		−1.79	0.61	−1.74	0.52	
Emotional intensity (1–5)	Public	3.56	0.97	3.49	0.90	0.50	3.41	0.96	3.69	0.88	0.76	3.66	0.84	3.30	1.05	1.43
	Private	4.60	0.63	4.42	0.81		4.45	0.76	4.60	0.66		4.58	0.67	4.40	0.80	
Psychosocial functions																
Self-continuity (1–5)	Public	1.80	0.87	1.96	0.98	0.06	1.68	0.83	2.17	0.99	7.53**	1.85	0.87	1.93	1.01	4.21*
	Private	2.65	1.04	2.85	0.99		2.72	1.02	2.80	1.02		2.84	0.97	2.60	1.08	
Social bonding (1–5)	Public	1.53	0.77	1.74	0.86	2.56	1.50	0.75	1.82	0.89	7.42**	1.69	0.87	1.54	0.73	3.86
	Private	2.28	1.13	2.24	1.08		2.31	1.19	2.20	0.98		2.43	1.12	1.98	1.03	
Directive (1–5)	Public	1.78	0.91	1.95	0.90	0.18	1.58	0.74	2.27	0.97	15.40***	1.87	0.93	1.85	0.88	2.80
	Private	2.56	1.02	2.67	0.89		2.57	0.97	2.68	0.94		2.72	1.01	2.44	0.83	

*N* = 233. *p < 0.05, **p < 0.01, ***p < 0.001.

Finally, 2 (public/private) × 2 (female/male) Mixed-Design ANOVAs were carried out on phenomenological dimensions. The phenomenological aspects of private and public FBMs revealed different patterns based on gender. While women rehearsed their private FBMs more than men did, men rehearsed public FBMs more than women did (*F*_1, 231_ = 12.23, *p* ≤ 0.001, *𝜂_p_^2^* = 0.05). Men reported that public FBMs produced more consequences for their lives in general, whereas women reported that their private FBMs produced more consequences for their lives (*F*_1, 231_ = 4.76, *p* < 0.05, *𝜂_p_^2^* = 0.02). The results of the comparisons of vividness (*F*_1, 231_ = 3.14, *p* = 0.08), importance (*F*_1, 231_ = 0.25, *p* = 0.62), surprise (*F*_1, 231_ = 0.41, *p* = 0.52), emotional valence (*F*_1, 231_ = 0.30, *p* = 0.58), and emotional intensity (*F*_1, 231_ = 1.43, *p* = 0.23) were not significant.

### 3.3 Psychosocial functions of the FBMs

Three psychosocial functions of the private and public FBMs were analyzed through one-way Repeated ANOVAs. Comparisons based on event type for each function, self-continuity (*F*_1, 232_ = 138.69, *p* < 0.001, *𝜂_p_^2^* = 0.37), social bonding (*F*_1, 232_ = 67.61, *p* < 0.001, *𝜂_p_^2^* = 0.23), and directive functioning (*F*_1, 232_ = 103.12, *p* < 0.001, *𝜂_p_^2^* = 0.31) showed that private FBMs were more functional for participants than public FBMs, with large effect sizes (see Table 4).

All three psychosocial functions of private and public FBMs for high and low SDO, SJT, and gender were analyzed separately using 2 × 2 Mixed-Design ANOVAs. Event type was included in the analysis as within-subject factors, and SDO, SJT, or gender were included in the analysis as between-subject factors in each analysis. Results for the SDO categories revealed no differences for the functions of private and public FBMs (self-continuity: *F*_1, 231_ = 0.06, *p* = 0.81; social bonding: *F*_1, 231_ = 2.56, *p* = 0.11; directive: *F*_1, 231_ = 0.18, *p* = 0.67). As for SJT, although public FBMs were perceived as more functional in terms of self-continuity, social bonding and directive functions in participants with high SJT than in those with low SJT (self-continuity: *F*_1, 231_ = 7.53, *p* < 0.01, *𝜂_p_^2^* = 0.03; social bonding: *F*_1, 231_ = 7.42, *p* < 0.01, *𝜂_p_^2^* = 0.03; directive function: *F*_1, 231_ = 15.40, *p* < 0.001, *𝜂_p_^2^* = 0.06), no significant differences were found between the high and low SJT groups for private FBM. Lastly, results for gender showed that, compared with men, women rated private FBMs as more functional in terms of self-continuity but men considered public FBMs as more functional in terms of self-consistency (*F*_1, 231_ = 4.21, *p* < 0.05, *𝜂_p_^2^* = 0.02). The results of the comparisons of social bonding (*F*_1, 231_ = 3.86, *p* = 0.05) and directive function (*F*_1, 231_ = 2.80, *p* = 0.09) were not significant (for means and standard deviations, see Table 5).

### 3.4 Participation in protest actions

One-way ANOVAs were conducted to examine the frequency of participation of high and low SDO and SJT and gender groups in protests against the coup attempt that took place afterward. Although no significant differences were observed in the level of participation in protest actions between genders (F_1, 231_ = 0.39, *p* = 0.53) and high and low SDO groups (*F*_1, 231_ = 0.001, *p* = 0.98), SJT was found to be a variable affecting participation in protest actions (*F*_1, 231_ = 35.60, p < 0.001, *𝜂_p_^2^* = 0.13): participants in the high SJT group (M = 1.87, SD = 0.76) took part in protest actions more than those in the low SJT group (M = 1.39, SD = 0.46).

## 4 Discussion

This study’s primary aim was to investigate whether the structural properties and functions of negative private and public FBMs vary depending on individuals’ SDO levels and beliefs about the social system. This study contributes to the existing literature by considering FBMs partly as a product of social construction influenced by a broader social context rather than as typical elements of autobiographical memory. As an alternative to the mainstream approach, which generally considers FBMs as individual memory elements that are relatively independent of social structures, there has been a growing tendency in recent years to examine them in relation to other human characteristics such as emotions, thoughts, and beliefs. However, these attempts have mostly lacked a theoretical framework to organize significant findings concerning societal dynamics, ranging from gender relations to intergroup tensions. The present study used the theories of intergroup relations (i.e., social dominance and system justification theories) for a multilevel perspective on the social factors that may influence the formation, maintenance, and use of FBMs, aiming to answer whether the structural and functional properties of FBMs in men and women with high and low SDO and SJT differ. Considering that SDO and SJT have only been used as variables in one cross-national study (Liu et al., 2021) on LHMs, this descriptive study provides preliminary evidence for extending the literature on the relationships between FBMs and group hierarchies.

In the present study, descriptive analyses of the canonical qualities of private and public FBMs indicated that *all* participants remembered the night of July 15, 2016. Given that gender, SDO, and SJT did not differ with regard to socioeconomic status, this can be seen as further evidence of the widespread importance of the coup attempt for the Turkish people. However, the canonical qualities exhibited a considerable variance in the sample. When participants were categorized into “weak” and “strong” FBM categories, approximately one-third of the sample was placed into the “strong” category. As the event occurred 3 years before data collection in this study, this result indicates the importance of this public event for young people in Türkiye. However, even in the face of this important public event, private FBMs presented a high level of canonical quality when statistically contrasted with public FBMs: private FBMs of participants were placed in the “strong” category more than public FBMs. This finding is consistent with previous studies showing the relative importance of private FBMs in people’s lives (Demiray and Freund, 2015; Pillemer, 2009).

When analyzed in terms of gender, SDO, and SJT categories, the canonical qualities of private and public FBMs presented some significant differences. First, regarding private FBMs, memory quality did not differ between gender groups, indicating the importance of these memories for individuals regardless of gender. However, FBM’s canonical quality of public FBM was higher for men, showing that men better coded the details of the night of the coup attempt. Since men reported more interest in following the political agenda, this memory advantage of male undergraduates may be due to the detailed information they encountered from several media outlets. There was no association between the high-and low-SDO categories and the canonical category qualities of both types of FBMs. The same result was obtained for the high-and low-SJT categories for the quality of public FBMs, showing no memory advantage for public FBMs in any of these categories. Apart from a marginally significant finding showing a difference between the high and low SJT groups concerning the relative superiority of high SJT participants in the quality of private FBMs, the results indicated that the high and low SDO and SJT groups did not differ significantly with regard to the canonical qualities of their private or public FBMs.

The findings above on canonical properties raise the question of whether the phenomenological and functional properties differ in the SDO or SJT categories. The within-subject analyses conducted with all participants showed that, apart from the surprising property, private FBMs had higher qualities than public FBMs regarding all phenomenological properties (importance, consequentiality, emotional intensity, vividness, rehearsal, and emotional valence). These findings align with those in the literature. Given the existing findings that private FBMs are more functional than public FBMs regarding self-continuity, social bonding, and directive functions (Demiray and Freund, 2015), it is not surprising that private FBMs have some advantages in being encoded in individuals’ memories, depending on their relevance and importance to individuals’ private lives. Perhaps more importantly, this study showed that this tendency is valid for all the main categories: individuals described their private FBM as more vivid, important, consequential, negatively emotional, and emotionally intense, regardless of gender, SDO, or SJT. The finding that public FBMs differ from private FBMs in terms of the surprise aspect can be seen as a clear sign of the extent to which the coup attempt caught individuals unprepared. This finding about the surprise property, which was also obtained in a previous study with a more heterogeneous sample (Çavuşoğlu et al., 2021), reveals again that this FBM constitutes a very suitable example of a public FBM for future studies to be realized in the Turkish context.

The between-subject analyses produced notable findings across the categories used in this study. Two phenomenological aspects revealed differences between gender groups: rehearsal and consequentiality. While women rehearsed their private FBM more often than men, men rehearsed their public FBM more often than women. In addition, men reported that public FBMs had more consequences for their lives than women did, whereas women rated their private FBMs as more consequential to their lives than men did. These findings suggest that this negative public FBM has a distinctive value for men and may reflect differentiation in the socialization processes of gender groups. Social dominance theory suggests that men are the main actors in conflicts and clashes in an arbitrary system in which young men are the main targets of violence and domination (Sidanius and Pratto, 1999). Therefore, it is plausible to conclude that FBMs related to public events that have the potential to change social hierarchy are considered more important by men in terms of the consequences they can produce in their lives. Because the SDO categories did not differ significantly in terms of phenomenological properties in either type of FBM, these findings indicate that SDO, an individual variable, may not be effective in FBMs. SDO, defined as the desire to maintain a social hierarchy, may not produce a particular tendency for individuals to differentiate between private and public FBMs.

However, the picture was clearer for the high and low SJT categories: while participants in these two categories did not differ in terms of the characteristics they attributed to their private FBMs, participants in the high SJT category perceived their public FBMs as more negative, important, and consequential than participants in the low SJT category. This difference may be because of the threatening nature of coup attempts for participants who support the status quo in the country. Given that high-SJT participants placed greater importance on their social identities than low-SJT participants, it seems possible to speculate that high-SJT participants perceived the coup attempt as a threat to their social identities and the status quo. Although the social identities of the participants were unknown, the finding that the high SJT group also had a more right-wing political orientation points to the possibility that these participants had social identities that were aligned with ideologies of conservatism and nationalism.

In parallel with the above findings, private FBM was more effective than public FBM in all psychosocial function types (self-continuity, social bonding, and directive functioning). These findings provide additional evidence of the particular importance of private FBMs for individuals’ lives compared to public FBMs (Demiray and Freund, 2015). However, this tendency was stronger for women than for men with regard to self-continuity function of private FBMs: these FBMs fulfilled self-continuity function to a higher degree for women than for men. On the other hand, in public FBM, the self-continuity function was more important for men. From the perspective of the arbitrary-set system of social dominance theory, it is understandable that public FBMs are more closely related to men’s status and the actions associated with their hierarchical positions, as the coup attempt threatened the status quo.

Again, no significant differences were observed in the psychosocial functions of FBMs between the high-and low-SDO categories. Another clear difference was observed in the SJT categories with regard to the functions of FBMs. While these categories did not differ in the functions of private FBM, participants in the high SJT category scored higher than participants in the low SJT category in all three functions of public FBM. The fact that public FBM fulfills many functions for individuals who legitimize the system to a great extent is among the important findings of this study.

The emergent picture is consistent with the findings above regarding the frequency of participation in protests against the coup attempt. Among the main categories analyzed in this study, gender and high/low SDO categories did not differ in participation in protest actions. However, a clear difference was observed between the SJT categories in this respect. Participants in the high SJT category were more likely to participate in activities and actions protesting the coup attempt than those in the low SJT group. This finding shows that the importance of public FBM is also reflected in behaviors. Similar to Bonnot and Krauth-Gruber’s (2018) findings, the participants who justified the system to a high degree (i.e., the high SJT group) behaved in a way consistent with their motivational inclinations by supporting the system using different means, ranging from posting on social media to participating in protests in the face of a severe threat (i.e., the coup attempt) defying the status quo.

In summary, this study shows that the tendency toward system justification is closely related to the quality and functional use of public FBMs. Participants who justified the status quo had high-quality public FBMs that fulfilled their needs (self-continuity, social bonding, and directives). However, SDO was found to be irrelevant in this regard. This finding on SDO may indicate the possibility that this individual-level variable of social dominance theory has no bearing on the formation, quality, and maintenance of negative private or public FBMs. Given that individuals with high SDO perceive the social world as a “competitive and cut-throat place” (Perry et al., 2013, p. 117) in which survival is the primary goal, SDO may be a relatively distal factor, at least in this age group, in shaping and remembering autobiographical memories such as FBM. Although SDO seems irrelevant, some explanations of social dominance theory on group-based hierarchies are thought to effectively place the phenomenon of FBM in group hierarchies of gender and arbitrary set. Compared to young women who had relatively weaker public FBMs, which they tend to use only for the self-continuity function, young men were more interested in the political agenda, had high-quality public FBMs, and used their public FBMs to fulfill their self-continuity functions.

## 5 Limitations and conclusion

This study has some limitations. First, participants were undergraduate students who were not selected or grouped according to their real (or actual) group memberships. Ironically, this limitation arose because of the importance and societal consequences of the coup attempt, which the present study uses as an example of public FBM in a Turkish context. The coup attempt shook up society in many ways. Soon after the event, many civilians and military personnel were arrested and prosecuted, and many of them were convicted because of their involvement in the coup or their affiliation with an organization (FETO) held responsible for organizing the coup attempt. Prosecutions and arrests are ongoing. This created an atmosphere in the following years in which people were reluctant to report their political affiliations or tendencies openly. This tendency was still high in the third year after the coup attempt, when the data collection for this research began. Therefore, we decided to refrain from asking specific questions about the political, religious, and ethnic group memberships or affiliations of the participants, which might make them suspicious of the aim of the research and reduce the possibility of participation. Instead, they were asked general questions about their political orientations and levels of commitment to their social identities (which were unknown to us). Although the present study was able to examine gender differences and similarities in FBMs in a theoretically informed way for the first time, the lack of knowledge on actual group memberships or affiliations has limited our ability to test other hypotheses of social dominance and system justification theories, as these theoretical perspectives drew primarily on actual group memberships. Therefore, future studies with samples from real groups will undoubtedly extend the scope of the existing literature.

Another limitation was the age group of the participants sampled in the present study. The participants were 17 years old on average when the coup attempt took place, which may have created an advantage for them in remembering this public event with its rich details and quality. Evidence shows that critical age-related differences in forming and recalling autobiographical memories, of which FBM is a subtype, are possible. Research indicates that events between the ages of 12 and 29 are more likely to be of higher quality and are recalled better by individuals. This phenomenon, called the “reminiscence bump,” is one of the most consistent findings in memory studies (Munawar et al., 2018, p. 2). Therefore, the age groups included in this study may limit the generalizability of the findings to other age groups.

Another age-related limitation was the lack of information on the dates of the participants’ private FBMs. While the dates of the participants’ public FBMs were obvious, no such information was asked of private FBMs to keep the number of volunteers for the main study as high as possible. However, it is clear from the within-subject analyses that this omission does not pose a problem for the study, as the well-known superiority of private FBMs over public ones is apparent in our results.

The findings of this study are limited by Türkiye’s political, social, and cultural conditions. As Liu et al. (2021) showed using the LHM, countries exhibit considerable variation in the relationships between the variables of SDO and SJT. Therefore, the present study’s findings must be compared to those of studies conducted in other countries. In addition, the descriptive nature of the present study prevents any attempt to discuss the causal relationships among the variables covered. Scholars in this line of research insist that creating FBMs through experimentation is the most effective method for this type of work (Lanciano et al., 2010, 2018b).

Finally, this study focused only on negative FBMs, whether private or public. This limits the scope of the study to the relationships between negative events and individual-level variables of SDO and SJT. Although it is not possible to speculate on their relationships with SDO, there is no doubt that positive FBMs, especially public ones, contribute significantly to individuals’ perceptions of the status quo and SJT.

However, despite its limitations, this descriptive study may pave the way for examining FBMs from the perspective of intergroup theories by answering the research questions asked at the beginning. Owing to their distinctive properties and functions, FBMs play an important role in forming and maintaining the collective memories of societies and groups (Cheriet et al., 2023; Hirst and Meksin, 2018). Memories are undoubtedly part of the repertoire that groups use when defining their shared realities. We posit that multilevel theories of intergroup relations provide many possibilities for discovering the dynamics ranging from the individual to systemic factors behind memory processes by combining attempts at bottom-up and top-down examinations of memories (Hirst et al., 2018), including FBM.

## Data Availability

The dataset presented in this study can be found at: https://osf.io/xbfkp.

## Footnotes

^1^Political orientation was measured on a seven-point scale ranging from −3 to +3 (*extreme, moderate, mild*) from left to right. Before the analyses, they were recoded from 1 (*extreme left*) to 7 (*extreme right*).

^2^All statistical analyses in the current study used pairwise comparisons to maximize the number of participants; therefore, the *N* values differ.
